# Comparative proteomic analysis of osteogenic differentiated human adipose tissue and bone marrow‐derived stromal cells

**DOI:** 10.1111/jcmm.15797

**Published:** 2020-09-03

**Authors:** Mehran Dadras, Caroline May, Johannes Maximilian Wagner, Christoph Wallner, Mustafa Becerikli, Stephanie Dittfeld, Bettina Serschnitzki, Lukas Schilde, Annika Guntermann, Christina Sengstock, Manfred Köller, Dominik Seybold, Jan Geßmann, Thomas Armin Schildhauer, Marcus Lehnhardt, Katrin Marcus, Björn Behr

**Affiliations:** ^1^ Department of Plastic Surgery BG University Hospital Bergmannsheil Bochum Germany; ^2^ Medizinisches Proteom‐Center Ruhr‐Universität Bochum Bochum Germany; ^3^ Department of General and Trauma Surgery BG University Hospital Bergmannsheil Bochum Germany

**Keywords:** bone, mesenchymal stem cells, osteogenesis, proteome, proteomics

## Abstract

Mesenchymal stromal cells are promising candidates for regenerative applications upon treatment of bone defects. Bone marrow‐derived stromal cells (BMSCs) are limited by yield and donor morbidity but show superior osteogenic capacity compared to adipose‐derived stromal cells (ASCs), which are highly abundant and easy to harvest. The underlying reasons for this difference on a proteomic level have not been studied yet. Human ASCs and BMSCs were characterized by FACS analysis and tri‐lineage differentiation, followed by an intraindividual comparative proteomic analysis upon osteogenic differentiation. Results of the proteomic analysis were followed by functional pathway analysis. 29 patients were included with a total of 58 specimen analysed. In these, out of 5148 identified proteins 2095 could be quantified in >80% of samples of both cell types, 427 in >80% of ASCs only and 102 in >80% of BMSCs only. 281 proteins were differentially regulated with a fold change of >1.5 of which 204 were higher abundant in BMSCs and 77 in ASCs. Integrin cell surface interactions were the most overrepresented pathway with 5 integrins being among the proteins with highest fold change. Integrin 11a, a known key protein for osteogenesis, could be identified as strongly up‐regulated in BMSC confirmed by Western blotting. The integrin expression profile is one of the key distinctive features of osteogenic differentiated BMSCs and ASCs. Thus, they represent a promising target for modifications of ASCs aiming to improve their osteogenic capacity and approximate them to that of BMSCs.

## 1 INTRODUCTION

The treatment of bone defects caused by infection, trauma or neoplasms remains a clinical challenge. Autologous bone transplantation is limited by availability of donor sites, with iliac crest being the most common, apart from donor site morbidity that restricts the size of transplants, as well as the surgical risk factors.^1^ This has given rise to stromal/stem cell‐based therapy.^2^ Adult mesenchymal stromal cells (MSCs) can be harvested from different tissues such as bone marrow, adipose tissue, dental pulp and other tissues.^3^ They have stem‐like properties and are able to undergo differentiation into different mature mesenchymal cell types, given certain conditions and stimuli.^4^ In 2006, the International Society for Cellular Therapy (ISCT) proposed minimum criteria for classification as mesenchymal stromal cells. They must be plastic‐adherent (eg to a tissue culture flask), express surface markers CD73, CD90 and CD105 (≥90%), lack haematopoietic lineage markers CD14, CD34, CD45, CD19 and HLA‐DR (≤2%) and should be able to differentiate into mesodermal lineage (osteogenic, adipogenic and chondrogenic).^5^ Lately, paracrine effects of MSCs have gained attention as an important mode of action, as exosomes represent a way of cell‐free regenerative therapy.^6^

Bone marrow‐derived stromal cells (BMSCs) have been studied to a large extent and show a high regenerative potential, although their use is still limited by availability of donor sites for bone marrow aspiration, morbidity of the procedure—although lower than for bone grafting^7^—and the relatively low cell yield, as they represent <0.1% of cells harvested from bone marrow aspirate.^8^, ^9^ At the same time, they are the closest and most obvious mesenchymal stromal cells for bone tissue engineering, given their tissue origin, and unlike other mesenchymal stromal cells their ability to support formation of haematopoietic marrow.^10^

Adipose tissue‐derived stromal cells (ASCs) as part of the stromal vascular fraction of adipose tissue can likewise undergo osteogenic differentiation and may be isolated in sufficient quantities from lipoaspirates after liposuction. Here, it has been shown that there are no major differences in regard to proliferation or differentiation capacity of ASCs derived from subcutaneous fat of different anatomical regions.^11^

It has been shown that BMSCs are more prone to senescence during expansion and passage and more affected by ageing in terms of proliferative capability than ASCs, while at the same time osteogenic differentiation capacity is reported to be the lineage least impacted by age.^12^, ^13^

Multiple studies have compared the characteristics of these two mesenchymal stromal cells in regard to bone tissue engineering in vitro. Most studies point to inferior extracellular matrix mineralization and lower expression of key osteogenic transcription markers like Runx2 in osteogenically differentiated ASCs compared to BMSCs.^14^, ^15^ An intraindividual comparison of human MSCs of three donors cultured on decellularized porcine bone confirmed superior osteogenic capacity of BMSCs compared to ASCs.^16^ On the other hand, a study by Rath et al found better osteogenic differentiation of ASCS compared to BMSCs using 3D bioglass scaffolds as a particular culturing condition.^17^

Brennan et al isolated human BMSC from bone marrow aspirates and ASCs from lipoaspirates of healthy donors and characterized the cells based on surface markers and tri‐lineage differentiation as outlined above. In an ectopic nude mouse model, BMSCs but not ASCs were able to induce ectopic bone formation.^18^ In a critical size defect model of sheep tibia, application of ovine BMSCs isolated from bone marrow aspirates resulted in a significantly higher amount of newly formed bone tissue than application of ovine ASCs isolated from excised subcutaneous fat tissue.^19^ Importantly, osteogenically differentiated ASCs do not support the formation of a hematopoietic marrow.^10^, ^20^

Proteomics enables large‐scale analysis of proteins present in a cell type in trying to gain mechanistic insight as to the underlying reasons for functional differences and can be used to identify differentially regulated key proteins in a comparative approach.

Roche et al performed a comparative proteomic analysis of human BMSCs and ASCs cultured in different laboratories and characterized by FACS analysis, with number of donors or isolation method not reported. They identified 556 proteins, with 78% of these not being differentially regulated between these two cell populations, which is regarded as high similarity.^21^ Another comparative proteomic study by Jeon et al 2016 used commercially available human BMSCs and human ASCs isolated from lipoaspirates or lipectomy specimens without reporting the number of donors, characterized by FACS analysis and tri‐lineage differentiation. They found 90 differentially regulated proteins out of 3000 identified proteins.^22^ However, both studies analysed undifferentiated MSCs. Focusing on osteogenic differences, we found only a transcriptomic comparison of osteogenically differentiated porcine ASCs and BMSCs by Monaco et al from 2012, which resulted in 21 differentially expressed genes after 21 days of osteogenic culture conditions.^23^ Giusta et al performed a proteomic analysis of human ASCs of three donors undergoing osteogenic differentiation and found 28 proteins that were differentially regulated between the undifferentiated state and after 4 weeks of osteogenic differentiation.^24^

To our knowledge, no comparative proteomic analysis of human ASCs and BMSCs after osteogenic differentiation has been performed to date. Thus, it still remains unanswered which key distinctive features of osteogenic differentiated ASCs and BMSCs at protein level might help address the abovementioned weaknesses of ASCs in bone tissue engineering/regeneration for translational research.

To overcome this need, an intraindividual comparative data‐independent acquisition (DIA)‐based proteomic analysis of osteogenic differentiated human BMSCs and ASCs was performed in this study.

## 2 MATERIALS AND METHODS

The study was approved by the ethics committee of the Ruhr University Bochum (Approval number: 5045‐14) and was conducted according to the Declaration of Helsinki. Written consent was obtained from all patients included in the study.

**FIGURE 1:**
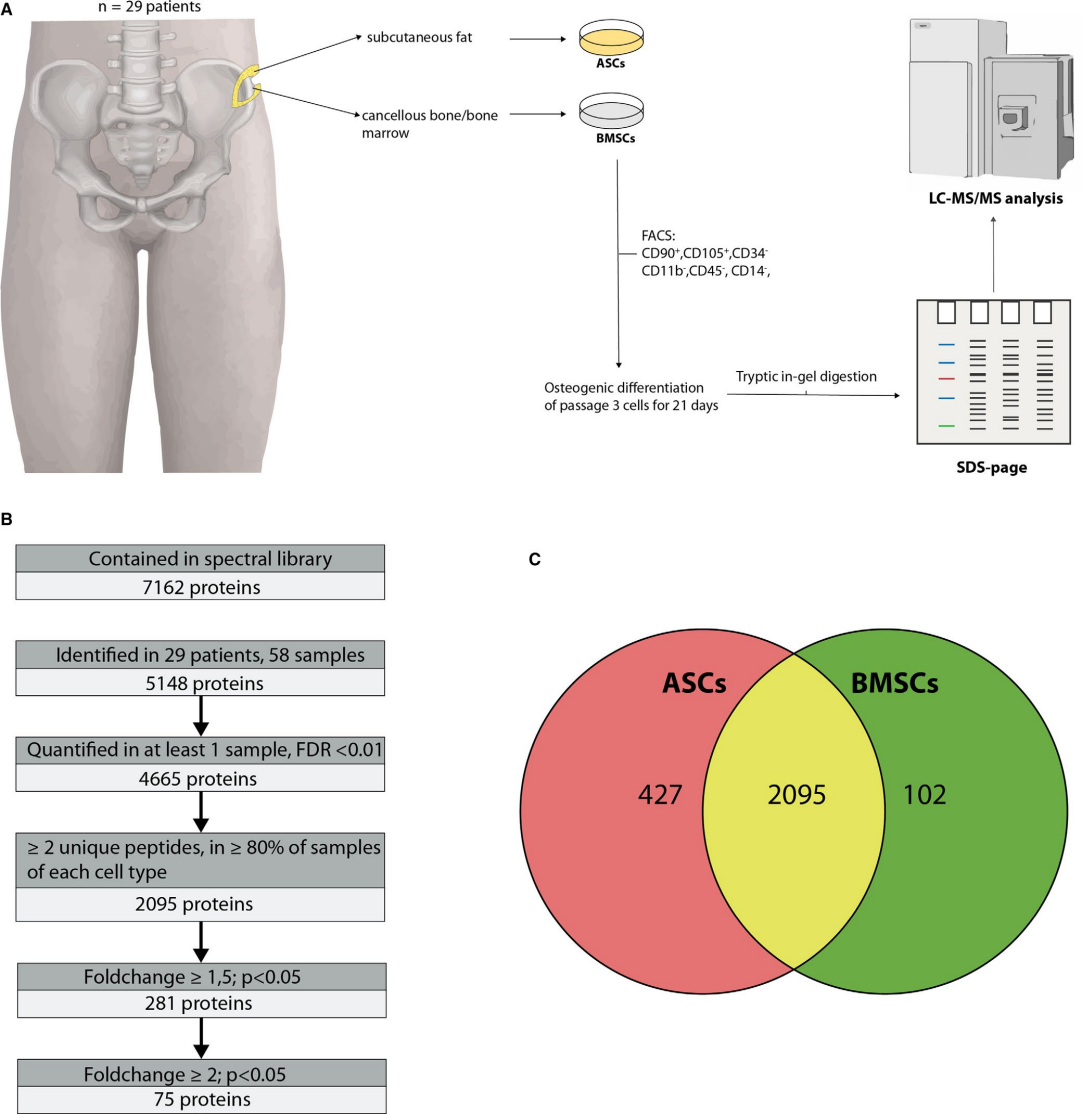
A, Graphical study setup. Harvest of cancellous bone and subcutaneous fat samples from patients undergoing autologous bone graft surgery and subsequent isolation of ASCs/BMSCs, osteogenic differentiation and proteome analysis. B, Flow chart of identified and filtered proteins. C, Venn diagram of proteins quantified in 80% of ASCs, BMSCs and both cell types

The study setup is illustrated in Figure 1A. In patients undergoing autologous bone transplantation from the iliac crest in the departments for trauma surgery and plastic surgery/hand surgery, cancellous bone that had been removed in excess and a small amount of subcutaneous fat from the surgical site at the iliac crest were harvested as paired samples. Patients aged 18‐89 were eligible as study participants. No exclusion criteria were applied.

### 2.1 Cell isolation

ASC and BMSC isolation was performed following modified standard protocols as described in our data article on the generation of the spectral library for this project.^25^, ^26^, ^27^, ^28^, ^29^ In brief, adipose tissue was rinsed with pre‐warmed (37°C) PBS (PAN Biotech, Germany). Blood vessels and connective tissue were carefully detached and discarded, before the tissue was minced. Samples were then weighed. 5 mL of collagenase IV (1 mg/mL, Cell Systems, Germany) per gram of tissue material was utilized for digestion. This was followed by incubation for 1 hour at 37°C and 330 rpm on a heated shaker. Afterwards, the reaction was stopped by addition of 25 mL base medium (DMEM/HAM's F‐12 [PAN Biotech, Germany], 10% foetal bovine serum, 1% penicillin/streptomycin).

Cancellous bone was flushed multiple times with base medium, and wash fractions were collected and combined.

Cell suspensions of both tissue sources were then filtered through a 100 µm cell strainer and centrifuged at 400 x *g* for 5 minutes at room temperature. Cell sediment was then resuspended in cold red blood cell (RBC) lysis buffer (1000 mL Milli‐Q‐water, 8 g ammonium chloride, 0.8 g sodium hydrogen carbonate and 0.4 g EDTA) and incubated on ice. Lysis was finished after 8 minutes by addition of base medium. Cells were again centrifuged at 400 x *g* for 5 minutes at room temperature. Cell sediment was resuspended in base medium, and cell concentration was measured utilizing the CASY^®^ Cell Counter (OLS OMNI Life Science, Germany). Cells were then plated in cell culture flasks according to their amount (10^6^ cells per T‐75 flask) and maintained in an incubator at 37°C and 5% CO_2_. 48 hours after plating, non‐adherent cells were washed off with pre‐warmed DPBS (PAN Biotech). Fresh base medium was added and replaced every 2 days.

### 2.2 Osteogenic differentiation

Passage three hASCs and hBMSCs were utilized for osteogenic differentiation.^18^ For this purpose, cells were washed and then detached using 0.05% Trypsin/EDTA (PAN Biotech, Germany). Cell suspension was centrifuged at 400 x *g* for 5 minutes at room temperature and the cell sediment resuspended in base medium. Cells were plated with a density of 2 × 10^5^ cells per 10 cm culture dish. Afterwards, osteogenic differentiation was induced by culturing the cells for 21 days in osteogenic differentiation medium consisting of the base medium, 10 mmol/L β‐glycerophosphate, 100 nmol/L dexamethasone and 250 µmol/L ascorbic acid. Then, cells were washed and mechanically detached cautiously using a cell scraper. Cells were collected and centrifuged at 160 x *g* for 5 minutes at 4°C. Supernatant was aspirated, and samples were stored at −80°C.

After 21 days culturing in osteogenic differentiation medium, extracellular matrix mineralization in hASCs and hBMSCs was examined. To this end, cells were carefully rinsed with DPBS and fixed with 4% paraformaldehyde (PFA) solution for 30 minutes at room temperature. Subsequently, cells were washed with distilled water and 2% Alizarin Red S solution (Sigma‐Aldrich, Germany) was added. Cells were incubated for 45 minutes in darkness, before the staining solution was removed and cells washed four times with distilled water.

### 2.3 Adipogenic differentiation

After preparation of cells in the same form as for osteogenic differentiation, adipogenic differentiation was induced by culturing cells for 21 days in base medium supplemented with 10 μg/mL Insulin, 1 μmol/L dexamethasone, 200 μmol/L indometacin and 0.5 mmol/L of 3‐isobutyl1‐methylxanthin (AppliChem, Germany).^30^ Afterwards, Oil red O staining was performed to visualize lipids in the vacuoles of the cells. Briefly, cells were washed twice with PBS, fixed with 10% formaldehyde for 10 minutes and then stained with Oil Red O solution (Sigma‐Aldrich) for 15 minutes. The cells were subsequently washed twice with distilled water.

### 2.4 Chondrogenic differentiation

For chondrogenic differentiation, cells were distributed in droplets to form spheroids. Chondrogenic differentiation was induced by culturing cells with the StemPro™ Chondrogenesis Differentiation Kit (Thermo Fisher Scientific, USA) for 21 days. Chondrogenic differentiation was assessed by staining spheroid cultures with Alcian Blue 8GX (Sigma‐Aldrich). Briefly, cells were washed twice with DPBS and fixed with 4% formaldehyde solution for 30 minutes. After fixation, cells were washed with DPBS and stained with 1% Alcian blue solution for approximately 12 hours. Afterwards, cultures were rinsed with 0.1 N hydrochloric acid and water.

### 2.5 Flow cytometry

Isolated and expanded cells at passage 3 were transferred to 5 mL tubes (BD Bioscience, Germany), and expression of cell surface markers CD90, CD105, CD34, CD11b, CD14 and CD45 (BD Pharmingen™, Germany) was analysed utilizing flow cytometry (FACSCalibur™, Becton Dickinson, USA). Data were analysed with CELLQuest™ 1.2.2 software (Becton Dickinson). Calibration reagents and solutions for flow cytometry were from Becton Dickinson. Once the appropriate instrument settings and compensations (FACSComp™ 2.0, Becton Dickinson) were achieved, instrument setup was not changed throughout the study. For each measurement, 10 000 cells were acquired, and regions of positive fluorescence were determined by the respective isotype control antibodies. Fluorescence signals of the isotype antibodies were adjusted as negative for 99% of gated cells.

### 2.6 Sample preparation for protein analysis

*Spectral library*—For creation of a spectral library, subcutaneous fat and cancellous iliac bone specimen of a healthy, non‐smoker, 24‐year‐old male patient was retrieved during an autologous bone transplantation procedure. Cell isolation, expansion and osteogenic differentiation were performed as described above. The methods for the creation of the spectral library are described elsewhere.^29^

*Patient specific samples—*The patient samples were prepared in the same way as the samples for the spectral library, followed by protein concentration determination by Bradford assay. These samples were used for data‐independent acquisition (DIA)‐based mass spectrometry as well as for Western blot analysis.

### 2.7 Data analysis of patient samples using the generated spectral library

Preparation of the patient specific samples for DIA‐based measurements was performed analogously to the samples for preparing the spectral library, with minor changes. 20 µg protein was loaded on the SDS gel, and electrophoresis was stopped after 15 minutes obtaining shorter gels compared to the approach described earlier for the spectral library. Afterwards, in‐gel trypsin digestion and peptide extraction were performed as described earlier. From the resulting peptide extract, 2 µL was used for determination of the peptide concentration by amino acid analysis, as described by Steinbach et al^31^ Samples were prepared for mass spectrometry, and 1 µL of iRT‐peptide (Biognosys AG, Switzerland) was added to each sample.

For mass spectrometric analysis, again a Q Exactive HF™ (Thermo Fisher Scientific Inc, USA) mass spectrometer was used and operated in DIA mode. The full MS1 scan ranged from 350 to 1200 m/z at a resolution of 120 000. Fragment ions were generated by HCD at a resolution of 30 000 and a stepped NCE of 25.5%, 27% and 30%, respectively. Default charge state was set to ≥+4, and first fixed mass was set to 200 m/z (ACG 1e6, maximum injection time 20 ms). The dataset has been uploaded to ProteomeXchange with the identifier PXD015223.

Data evaluation was carried out with the interface of Spectronaut™ Pulsar under standard settings. In short, the spectral library generated here was taken as a reference database and false discovery rate (called Qvalue) was set to a threshold of 1%. Proteins that could be quantified in at least 80% of samples from each cell type were used for further statistical evaluation. As additional filter criteria, fold changes (>50%) and adjusted *P*‐values (<5%) based on the Benjamini‐Hochberg method were calculated manually.

### 2.8 Western blot analysis of patient specific samples

Immunoblotting was performed, as described earlier for GFAP detection by Kurz et al,^32^ with minor changes. 50 µg of protein lysate was separated using 10% Bis‐Tris gels according to manufacturer's recommendations (Life Technologies, Germany). Proteins were transferred to nitrocellulose membranes using the iBlot transfer system (Thermo Fisher Scientific) followed by incubation in StartingBlock™ (Pierce, Woburn, USA) for 30 minutes and subsequent probing with primary antibody for 2 hours. For this, the primary antibodies were diluted in 50% TBS buffer/50% StartingBlock™. To remove unbound primary antibodies, the nitrocellulose membrane was washed three times for 10 minutes before incubation, followed by incubation with the fluorescent secondary antibody in 50% TBS buffer/50% StartingBlock™ for 1 hour. Finally, the membrane was washed in TBS buffer three times for 10 minutes. The Odyssey™ system (LI‐COR Biosciences GmbH, Germany) was used for fluorescence read out.

Anti‐ITGA3 rabbit polyclonal (ab190731, 1µg/ml), anti ITGA5 rabbit monoclonal (ab150361, 1:5000), anti‐ITGA7 rabbit polyclonal (ab182941, 0,5µg/ml) and anti‐ITGA11 rabbit polyclonal antibody (ab198826, 1:200) were obtained from Abcam, United States. Anti β‐actin (A228, Sigma‐Aldrich) secondary anti‐mouse IRDye800CW (1:15 000) was purchased from LI‐COR.

### 2.9 Pathway analysis

Pathway analysis was performed for all proteins up‐regulated ≥1.5 fold in BMSCs, using the Reactome database.^33^ In order to reference the number of up‐regulated proteins per pathway, the number of quantified proteins for each pathway was also determined.

## 3 RESULTS

The study involved 30 donor patients, after one patient being excluded due to insufficient yield in the process of protein isolation. Samples of one patient were used to generate a spectral library, while analyses were performed on samples of 29 patients. Median age was 52 (range 22‐85), and 21 of the patients were male. Mean BMI was 28.7 ± 5, and 15 of the patients were smokers. Patient characteristics are presented in Table 1.

**TABLE 1:** Study population

	Patients (n = 30)
Age	
<30 y	5 (17%)
30‒60 y	15 (50%)
>60 y	10 (33%)
Sex	
Male	20 (67%)
Female	10 (33%)
BMI	
18‐25	7 (23%)
>25	11 (37%)
>30	12 (40%)
Smoker	
Yes	15 (50%)
No	15 (50%)
ASA‐Classification	
1	3 (10%)
2	18 (60%)
3	8 (27%)
4	1 (3%)

Abbreviations: ASA‐Stage, American Society of Anesthesiologists; BMI, body mass index.

### 3.1 Identification of ASCs/BMSCs

**FIGURE 2:**
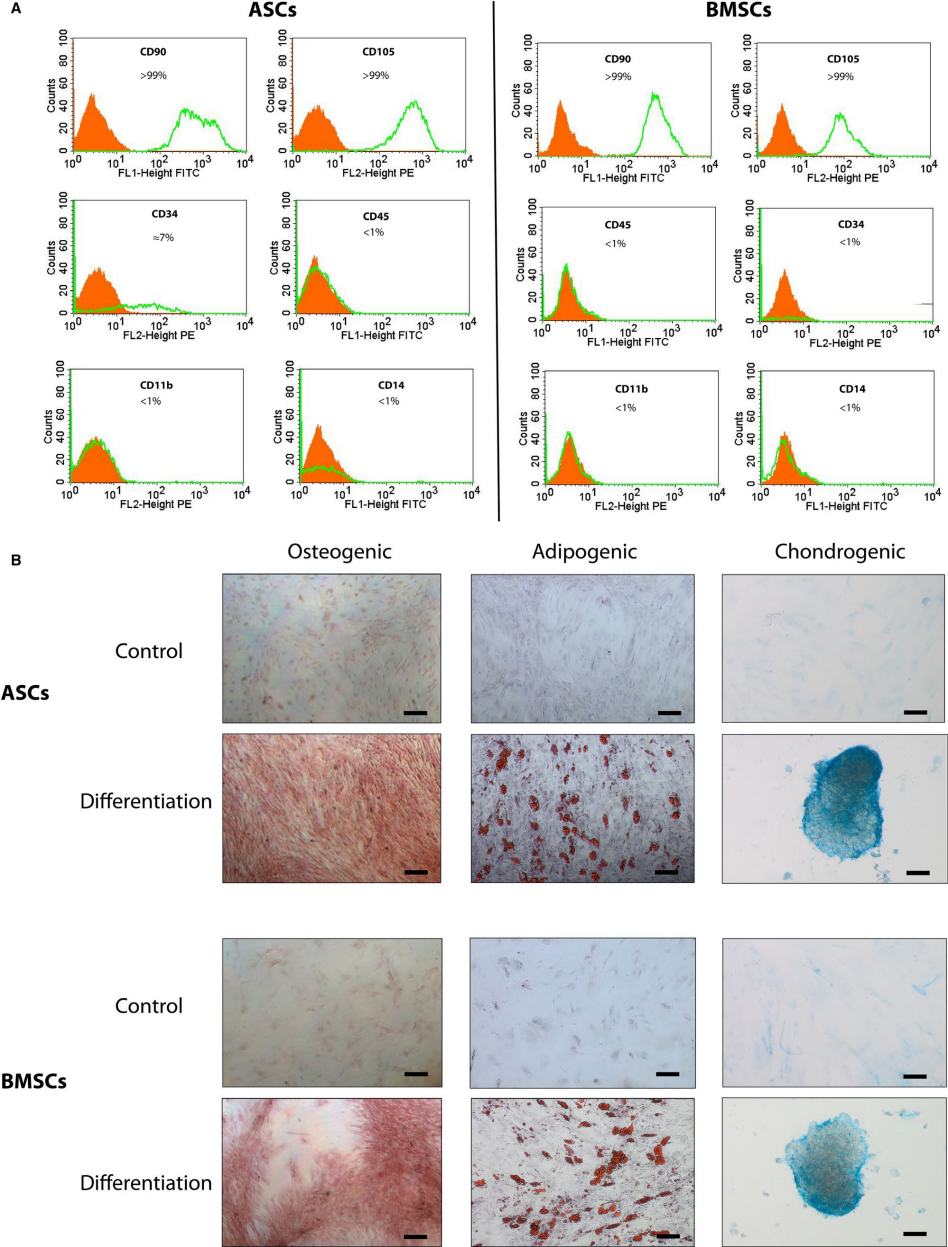
Identification of MSCs. A, FACS analysis of ASCs and BMSCs. Isotype controls are marked in orange. Expression of examined surface markers, which are demonstrated in the green line, meets MSC‐requirements. B, Alizarin Red staining of cells after 21 d of osteogenic differentiation, Oil red O staining of cells after 21 d of adipogenic differentiation and Alcain blue staining of cells after 21 d of chondrogenic differentiation. Scale bar: 100 μm

To characterize the hASCs and hBMSCs (passage 3), flow cytometry was used to identify the expression of different cell surface markers. Here, expression of typical mesenchymal stem cell markers such as CD90 and CD105 and lack of expression of haematopoietic cell surface markers such as CD14, CD11b, CD34 and CD45 were analysed. Figure 2A represents typical flow cytometry histograms. As shown, >99% of both cell types were positive for CD90 and CD105 and negative for CD14, CD45 and CD11b. While >99% of BMSCs were negative for CD34, around 7% of ASCs were CD34‐positive. Tri‐lineage differentiation was performed in both cell types and confirmed by Alizarin Red staining for osteogenic differentiation, Oil Red O staining for adipogenic differentiation and Alcain Blue staining for chondrogenic differentiation (Figure 2B). Cells of 6 random patients were characterized as described before and deemed representative of the whole population and the cell isolation and expansion protocols.

### 3.2 Comparative proteomic analysis

Of the patients, a 24‐year‐old healthy non‐smoker with no medical history who had undergone bone grafting for treatment of a scaphoid non‐union was chosen as reference patient to create a spectral library for DIA‐based proteomic analysis of cell samples. Here, 96 546 peptides were identified, which could be assigned to 7162 proteins. This patient was not included in further comparative analysis. A flowchart of the filtering process is presented in Figure 1B.

In 58 samples from 29 study patients, 5148 proteins could be identified. Only 2624 proteins that were identified by at least 2 unique peptides and that were present in at least 80% of samples of at least one cell type (ASCs or BMSCs) were included for further quantitative analysis. Of these, 2095 were quantified in ≥80% of both ASCs and BMSCs, 427 were exclusively quantified in ≥80% of ASCs and 102 were exclusively quantified in ≥80% of BMSCs, as demonstrated by the Venn diagram in Figure 1C. The 2095 proteins quantified in ≥80% of both ASCs and BMSCs are reported in supplementary Table 1, along with their abundance.

The 427 proteins quantified in ≥80% of ASCs only and 102 proteins quantified in ≥80% of BMSCs only are reported in supplementary Table 2. Of these, the 10 proteins with the highest mean abundance per cell type are presented in Table 2.

**TABLE 2:** Top 10 proteins quantified in ≥80% of samples of only one cell type sorted by decreasing mean abundance

rank	BMSCs		ASCss	
	Uniprot ID	Protein name	Uniprot ID	Protein name
1	P02649	Apolipoprotein E	G3V5Z3	Serine/threonine‐protein phosphatase 4 regulatory subunit 3A
2	P27658	Collagen alpha‐1(VIII) chain	P27487	Dipeptidyl peptidase 4
3	O94875	Sorbin and SH3 domain‐containing protein 2	P00325	Alcohol dehydrogenase 1B
4	Q12981	Vesicle transport protein SEC20	O43895	Xaa‐Pro aminopeptidase 2
5	P51911	Calponin‐1	F8WJN3	Cleavage and polyadenylation‐specificity factor subunit 6
6	O43854	EGF‐like repeat and discoidin I‐like domain‐containing protein 3	Q8IVF2	Protein AHNAK2
7	P19320	Vascular cell adhesion protein 1	A0A0C4DFV9	Protein SET
8	P48357	Leptin receptor	P11586	C‐1‐tetrahydrofolate synthase, cytoplasmic
9	Q6WCQ1	Myosin phosphatase Rho‐interacting protein	Q8NCA5	Protein FAM98A
10	P31513	Dimethylaniline monooxygenase [N‐oxide‐forming] 3	Q9BS40	Latexin

Comparing the abundance of 2095 proteins quantified in ≥80% of both cell types revealed 281 proteins with a fold change of at least 1.5 and statistical significance after application of a Benjamini‐Hochberg correction with a 5% false detection rate. Of these, 204 were more abundant in BMSCs while 77 were more abundant in ASCs.

### 3.3 Functional and pathway analysis

**FIGURE 3:**
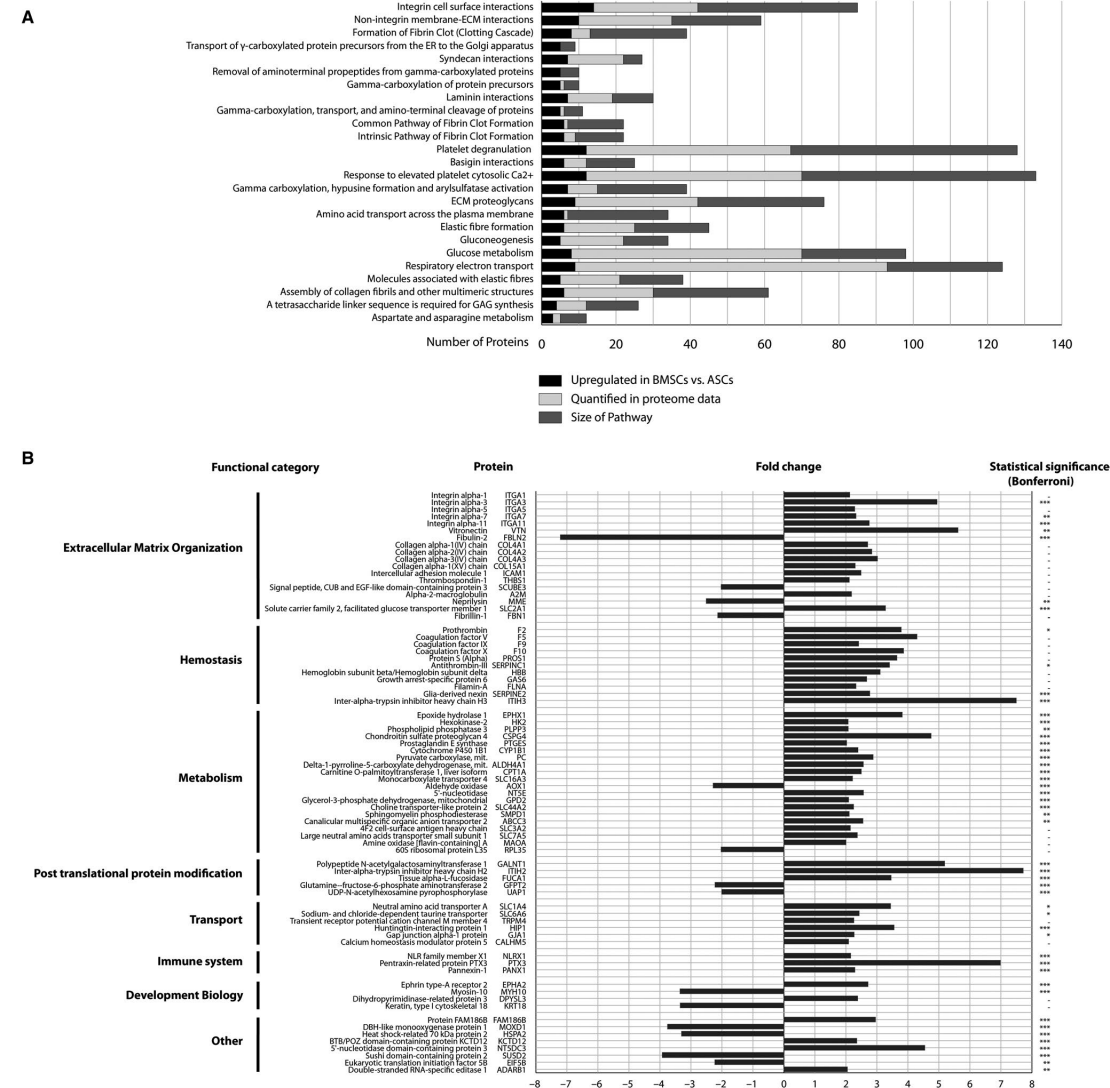
A, Reactome analysis. Results of Reactome overrepresentation pathway analysis of 204 proteins up‐regulated in BMSCs in order of statistical significance. Extracellular matrix/cell interactions are among the highest overrepresented pathways. B, Differentially expressed proteins. 75 proteins with a fold change ≥2 and corrected significance of <0.05 after application of a Benjamini‐Hochberg correction with a 5% false detection rate; of these 14 were more abundant in ASCs and 61 more abundant in BMSCs. Statistical significance is presented for a more strict Bonferroni correction. P‐value: *P < 0.05, **P < 0.01 and ***P < 0.001

Results of Reactome overrepresentation pathway analysis of the 204 proteins with higher abundance (fold change ≥1.5, *P* < 0.05 after Benjamini‐Hochberg correction with a 5% false detection rate) in BMSCs are presented in Figure 3A. Integrin cell surface interaction was statistically the most overrepresented pathway, with 14 more abundant proteins in BMSCs versus 43 total proteins in the pathway and 28 of them quantified in this study. Among the other highly overrepresented pathways are non‐integrin membrane‐ECM interactions, syndecan interactions, laminin interactions and basigin interactions, as other pathways of extracellular matrix/cell interaction. Statistically, most overrepresented pathways in pathway analysis of the 77 proteins with higher abundance in ASCs were biological oxidation, nucleobase biosynthesis and vitamin and cofactor metabolism.

75 proteins had a fold change >2 and statistical significance after application of a Benjamini‐Hochberg correction with a 5% false detection rate; of these, 14 were more abundant in ASCs and 61 more abundant in BMSCs. These proteins, along with the fold change, their functional affiliation and statistical significance level after application of a strict Bonferroni correction, are presented in Figure 3B. Of such proteins, 18 are affiliated with extracellular matrix organization, with 14 of these being more abundant in BMSCs. Among the ones with *P* < 0.001 after Bonferroni correction are integrin alpha‐3, integrin alpha‐11, fibulin‐2 and solute carrier family 2, facilitated glucose transporter member 1. Furthermore, 11 of the proteins are affiliated with haemostasis and 20 with metabolism.

**FIGURE 4:**
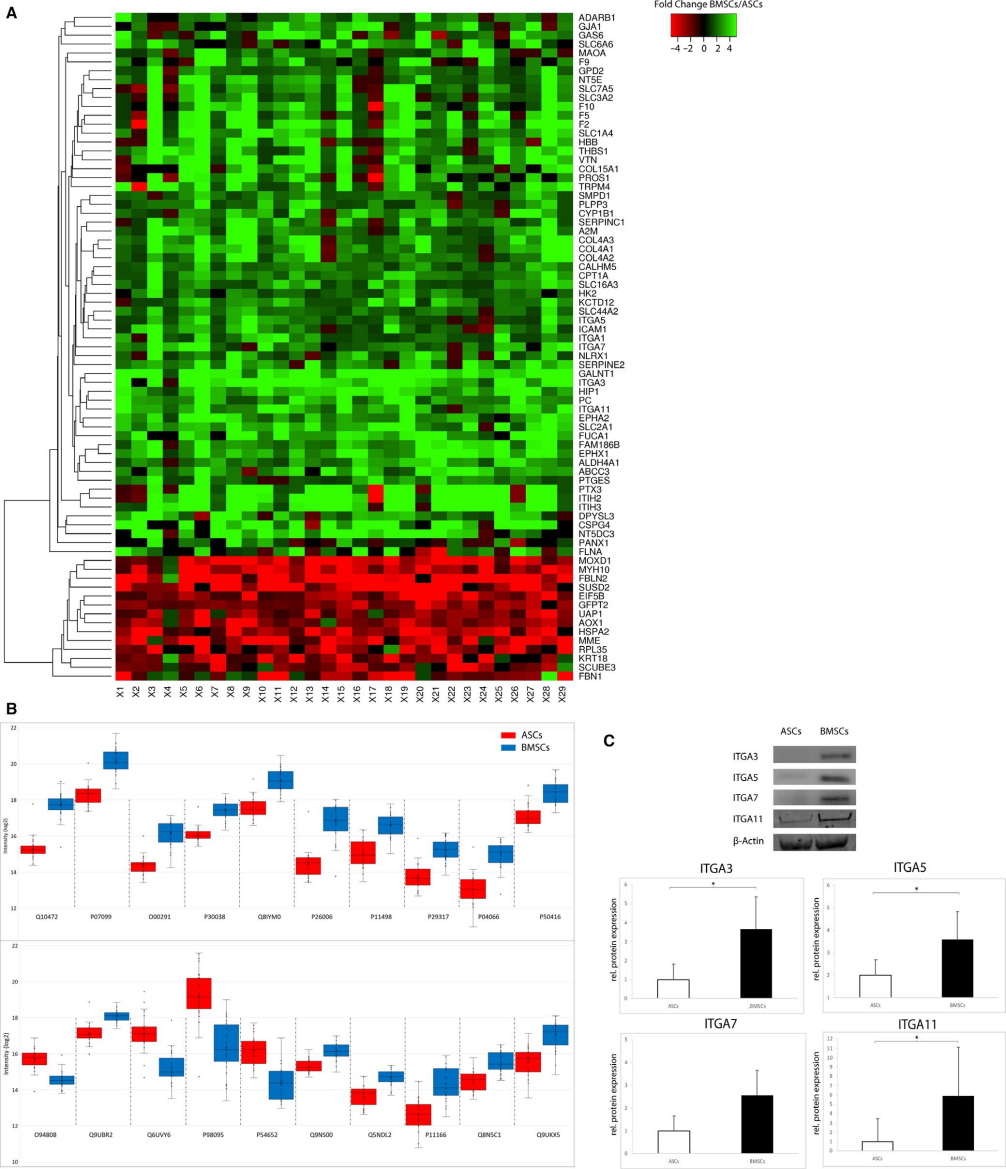
A, Intraindividual fold changes of differentially expressed proteins. Heatmap of intraindividual fold changes between BMSCs and ASCs for all proteins of Figure 4B and all 29 patients. B, Top 20 differentially expressed proteins. Boxplot of 20 proteins with highest fold change. Corresponding data are presented in Table 3. C, Western blot analysis. Western blot analysis of ITGA3, ITGA5, ITGA7 and ITGA11 in ASCs and BMSCs of six sample pairs confirms the results of the proteomic analysis, with significantly higher levels of integrins in BMSCs

A heatmap illustrating the fold change of these 75 proteins in each of the 29 patients is presented in Figure 4A, showing mostly homogenous fold changes in abundance between different patients.

The abundance of the 20 proteins with the highest fold change is presented as boxplots in Figure 4B, with the corresponding data in Table 3.

**TABLE 3:** Top 20 differential proteins by statistical rank in comparison of ASCs with BMSCs. Intensity shown as log_2_

Statistical rank	Uniprot ID	Protein name	Intensity ASCS	Intensity BMSCS
1	Q10472	Polypeptide N‐acetylgalactosaminyltransferase 1	15.29	17.72
2	P07099	Epoxide hydrolase 1	18.26	20.16
3	O00291	Huntingtin‐interacting protein 1	14.36	16.14
4	P30038	Delta‐1‐pyrroline‐5‐carboxylate dehydrogenase, mitochondrial	16.07	17.42
5	Q8IYM0	Protein FAM186B	17.56	19.12
6	P26006	Integrin alpha‐3	14.45	16.77
7	P11498	Pyruvate carboxylase, mitochondrial	15.04	16.61
8	P29317	Ephrin type‐A receptor 2	13.72	15.24
9	P04066	Tissue alpha‐L‐fucosidase	13.07	14.98
10	P50416	Carnitine O‐palmitoyltransferase 1, liver isoform	17.10	18.42
11	O94808	Glutamine‐fructose‐6‐phosphate aminotransferase [isomerizing] 2	15.70	14.57
12	Q9UBR2	Cathepsin Z	17.12	18.10
13	Q6UVY6	DBH‐like monooxygenase protein 1	17.10	15.21
14	P98095	Fibulin‐2	19.25	16.35
15	P54652	Heat shock‐related 70 kD protein 2	16.20	14.38
16	Q9NS00	Glycoprotein‐N‐acetylgalactosamine 3‐beta‐galactosyltransferase 1	15.32	16.14
17	Q5NDL2	EGF domain‐specific O‐linked N‐acetylglucosamine transferase	13.66	14.70
18	P11166	Solute carrier family 2, facilitated glucose transporter member 1	12.64	14.34
19	Q8N5C1	Calcium homeostasis modulator protein 5	14.47	15.53
20	Q9UKX5	Integrin alpha‐11	15.54	17.01

Western blot analysis of integrins alpha 3, alpha 5, alpha 7 and alpha‐11 as functionally relevant proteins with a highly significant difference in regulation was performed to validate the results of proteomic analysis. Here, significantly higher protein levels of all 4 Integrins in BMSCs compared to ASCs could be confirmed; results are shown in Figure 4C.

## 4 DISCUSSION

The aim of this study was to utilize intraindividual comparative proteomics to identify key proteins that are differentially expressed between ASCs and BMSCs and are potential candidates for improvement of the osteogenic potential of ASCs. We were able to successfully isolate cells from subcutaneous fat tissue and cancellous bone which are plastic adherent, show tri‐lineage differentiation and are CD90^+^, CD105^+^, CD11b^−^, CD14^−^, CD34^−^ and CD45^−^. These cells were therefore referred to as either ASCs or BMSCs. The small fraction of adipose‐derived cells that expressed CD34 (≈7%) is not in conflict with the supposed identification as mesenchymal stromal cells, as, contrary to the original minimum criteria defined by the International Society for Cellular Therapy,^5^ newer studies have pointed out that ASCs are CD34 positive to a certain extent but may lose this marker in the process of cultivation and expansion.^34^, ^35^ CD73 was not analysed but all other surface markers and the differentiation potential clearly indicated properties of mesenchymal stromal cells.

Comparative analysis of quantified proteins in ASCs and BMSCs revealed a number of proteins that were predominantly present in only one of the cell types. Apolipoprotein E, a protein primarily responsible for lipid metabolism, was quantified in >80% of BMSCs but not in ASCs. It has been shown in previous studies that apolipoprotein E expression is strongly induced upon differentiation and mineralization of osteoblasts.^36^ Further studies have confirmed its role as a regulator for osteoblastogenesis and osteoclastogenesis in mice.^37^, ^38^, ^39^

Another protein quantified in >80% of BMSCs but not in ASCs was leptin receptor, which has been identified as a distinct marker of bone marrow mesenchymal stromal cells responsible for bone formation.^40^ Leptin receptor–positive cells are the major source of bone and adipocytes in adult bone marrow.^41^ Detection of this protein in the majority of studied BMSCs underscores the correct identity of the cells. Nevertheless, leptin receptor signalling in BMSCs has been shown to inhibit osteogenesis and promote adipogenesis.^42^

In our analysis, 17 out of the 75 differentially regulated proteins with a fold change of at least 2 were affiliated with extracellular matrix organization. Accordingly, pathways of extracellular matrix/cell interaction were among the most overrepresented in pathway analysis, and integrin cell surface interaction was the one most significantly overexpressed pathway. Shaik et al very recently demonstrated up‐regulation of integrins alpha 10, 4, 7, E and 3 and beta 2, 8, L1 and 4 during osteogenic differentiation of human ASCs on a transcriptome level.^43^

The requirement of integrins for bone formation and differentiation of osteoblasts has consistently been demonstrated.^44^, ^45^ Gronthos et al reported differential growth of BMSCs on different extracellular matrix proteins mediated by integrins as extracellular matrix receptors.^46^ Also, the integrin expression profile changes during osteoblast differentiation, representing a crucial step in bone development.^47^

Integrin α5 (ITGA5), which was up‐regulated in BMSCs versus ASCs in our analysis, has been shown to be up‐regulated upon osteogenic differentiation of BMSCs and promote osteogenesis via IGF2 and IGFBP2.^48^, ^49^ In further research, peptide‐mediated activation of ITGA5 led to a marked increase of osteogenic markers in murine mesenchymal cells in vitro and induced increased bone formation upon injection in cranial bone in vivo.^50^ In a study by Srouji et al, lentiviral activation of ITGA5 led to improved healing of murine critical size bone defects treated with human BMSCs.^51^ In contrast, Di Maggio et al reported decreased bone formation for unpassaged human ASCs upon peptide activation of integrin α5β1 after seeding in hydroxylapatite scaffolds and implantation in nude mice.^52^

Integrin β1 itself has also been shown to be essential for osteoblast mineralization in mice.^53^, ^54^ Integrin α9β1 has been shown to mediate osteogenic effects of fibrinogen by Runx2 activation.^55^

While there were a total of 5 different alpha integrins that were up‐regulated in BMSCs compared to ASCs, integrin alpha‐11 (ITGA11) showed a 2.76‐fold change and was highly significant even after Bonferroni correction and was thus noteworthy. ITGA11 is one of the main mediators of cell adhesion of MSCs to collagen I and is up‐regulated upon osteogenic differentiation. Its silencing leads to a marked decrease in MSC survival and in focal adhesion kinase activity.^56^ Also, deficiency of integrin α2 and α11 leads to dwarfism with functional impairment of bone and systemic decrease in insulin‐like growth factor concentration.^57^ ITGA11 has only recently been identified as a receptor of osteolectin, an osteogenic growth factor that also has been discovered recently, and is required for maintenance of adult skeleton and osteogenic potential of BMSCs.^58^, ^59^ It has been shown that ITGA11 signalling activates the canonical Wnt pathway, and blockage of the latter also annihilates the osteogenic effect of osteolectin.^58^ While up‐regulation of multiple integrins has been found in transcriptome analysis of human ASCs upon osteogenic differentiation, ITGA11 was not up‐regulated.^43^ How altering ITGA11‐expression in ASCs affects their osteogenic potential is part of an ongoing study.

While multiple approaches to improve osteogenic potential of ASCs have been undertaken, one such is hypoxic preconditioning, which has been shown to improve proliferation and osteogenesis.^60^ Remarkably, a study on BMSCs similarly demonstrated positive effects of hypoxia on proliferation and stemness and was able to show an induced change in expression profile of integrins with up‐regulation of alpha integrins 1, 3, 5, 6, 11, V and beta integrins 1 and 3.^61^ Taken together, these results suggest that hypoxic conditioning of ASCs might very well involve integrin up‐regulation as a method of action to improve osteogenic capacity.

The results of our study depend on the identity and treatment of the used cells. The isolation and differentiation protocols used are common in the literature and all analysed cells were in identical passages to improve comparability, as passaging undeniably has an influence on cell characteristics and surface markers, the latter being confirmed by FACS analysis. Although our study cohort is heterogenous in terms of age, morbidity or cofactors like smoking which has been shown to affect mesenchymal stem cell function,^62^ it represents a typical cohort of patients needing autologous bone tissue transfer and thus being candidate for future regenerative applications. Also, the intraindividual comparison approach focuses on changes regardless of confounding interindividual factors.

This study has high statistical power, given the inclusion of 30 patients and identification of more than 7000 proteins. We were able to identify integrin expression profile as one of the key differentiators between osteogenically differentiated ASCs and BMSCs, and indicate its functional relevance, in the context of previous studies and the present literature.

In this study of intraindividual proteomic analysis of osteogenic differentiated human ASCs and BMSCs, we were able to identify integrin expression profile as one of the key differentiators. Further research is needed to investigate the role of integrins in general, and particularly integrin α 11, in osteogenesis of ASCs, and their potential as therapeutic targets to approximate osteogenic capacity of ASCs to that of BMSCs.

## CONFLICT OF INTEREST

The authors have no potential conflicts of interest to report.

## AUTHOR CONTRIBUTIONS

**Mehran Dadras:** Conceptualization (equal); Data curation (equal); Formal analysis (equal); Investigation (equal); Writing‐original draft (lead); Writing‐review & editing (equal). **Caroline May:** Conceptualization (equal); Data curation (equal); Formal analysis (equal); Investigation (equal); Methodology (equal); Writing‐original draft (equal); Writing‐review & editing (equal). **Johannes Maximilian Wagner:** Formal analysis (equal); Writing‐original draft (equal); Writing‐review & editing (equal). **Christoph Wallner:** Data curation (equal); Formal analysis (equal); Writing‐review & editing (equal). **Mustafa Becerikli:** Data curation (equal); Formal analysis (equal); Writing‐original draft (equal); Writing‐review & editing (equal). **Stephanie Dittfeld:** Data curation (equal); Writing‐review & editing (equal). **Bettina Serschnitzki:** Data curation (equal); Formal analysis (equal); Writing‐review & editing (equal). **Lukas Schilde:** Data curation (equal); Formal analysis (equal); Writing‐review & editing (equal). **Annika Guntermann:** Data curation (equal); Formal analysis (equal); Writing‐review & editing (equal). **Christina Sengstock:** Data curation (equal); Formal analysis (equal); Investigation (equal); Writing‐original draft (equal); Writing‐review & editing (equal). **Manfred Köller:** Data curation (equal); Formal analysis (equal); Writing‐original draft (equal); Writing‐review & editing (equal). **Dominik Seybold:** Resources (equal); Writing‐review & editing (equal). **Jan Geßmann:** Resources (equal); Writing‐review & editing (equal). **Thomas Armin Schildhauer:** Resources (equal); Writing‐review & editing (equal). **Marcus Lehnhardt:** Project administration (equal); Resources (equal); Writing‐review & editing (equal). **Katrin Marcus:** Data curation (equal); Formal analysis (equal); Methodology (equal); Visualization (equal); Writing‐review & editing (equal). **Björn Behr:** Conceptualization (equal); Project administration (equal); Writing‐original draft (equal); Writing‐review & editing (equal).

## Supporting information

Table S1

Table S2

## Data Availability

The proteomic dataset has been uploaded to ProteomeXchange with the identifier PXD015223. Tables S1 And S2 contain all proteins quantified in ≥80% of both cell types and all proteins quantified in ≥80% of only one cell type, respectively. Any additional data are available upon reasonable request.
